# Long Term Follow Up of Celiac Disease—Is Atherosclerosis a Problem?

**DOI:** 10.3390/nu6072718

**Published:** 2014-07-21

**Authors:** Anna Rybak, Bożena Cukrowska, Jerzy Socha, Piotr Socha

**Affiliations:** 1 Department of Gastroenterology, Hepatology and Nutrition Disorders, The Children’s Memorial Health Institute, Warsaw, 04-730, Poland; E-Mails: j.socha@czd.pl (J.S.); p.socha@czd.pl (P.S.); 2 Department of Pathology, The Children’s Memorial Health Institute, Warsaw, 04-730, Poland; E-Mail: b.cukrowska@czd.pl

**Keywords:** celiac disease, atherosclerosis, obesity, diabetes mellitus

## Abstract

Celiac disease (CD) is a lifelong condition and it often involves impaired nutrition, wide spectrum of symptoms and it requires constant dietetic treatment. The impact of the gluten-free diet on patients’ nutritional status and on the other biochemical parameters is being widely investigated. In this article we looked into particular risk factors that might lead to increased prevalence of atherosclerosis in CD patients, including nutritional status, gluten-free diet, lipids profile and concomitant disease—type 1 diabetes mellitus. Here, we present the current data and research on these risk factors of atherosclerosis with respect to celiac disease.

## 1. Introduction

Celiac disease (CD) is a systemic autoimmune complex genetic disease, with the prevalence of 0.3%–2% in the Western populations [1,2], characterized by intolerance against gluten peptides, resulting in combination of different, gluten-dependent symptoms, enteropathy, as well as appearance of specific auto-antibodies in HLA-DQ2, or DQ8-positive individuals. In the last two decades the clinical picture of CD has changed from the classic presentation with the predominance of gastrointestinal symptoms like steatorrhoea, abdominal pain, failure to thrive or malabsorpion, to a more atypical or silent form. CD is a chronic condition, requiring life-lasting treatment based on the gluten-free diet (GFD).

As the disease involves inflammation processes, specific diet and often impaired nutritional status, a question arises concerning its long term impact on patients’ health and concomitant diseases. Metabolic syndrome was defined as a cluster of easily measurable factors of cardiovascular risk [3]. The end-point, which is cardiovascular disease, can be easily assessed in adults and recently similar definition of metabolic syndrome was applied for children. It includes central obesity, insulin resistance and lipid disturbances [4]. Lipid screening was also advised for early detection of cardiovascular risk in children from high risk groups. As cardiovascular disease appears to be an inflammatory process leading to endothelial damage [5], several factors are additionally regarded to describe cardiovascular risk, including pro-inflammatory cytokines and CRP. Adipocytokine has been claimed to be a good marker of the metabolic syndrome in children [6]. Sedentary behavior is an independent factor which should be taken into account and physical activity can improve arterial function [7]. In 2004, West and colleagues studied 3790 adults with CD with respect to the diagnosis of hypertension, hypercholesterolaemia, atrial fibrillation, myocardial infarction and stroke [8]. They showed a lower prevalence of hypertension and hypercholesterolaemia in adults with CD compared with the general population. One year earlier, the same authors showed that total cholesterol is lower in patients with undetected CD assessed by endomysial antibodies in comparison to endomysial-negative controls [9]. In 2008, Wei *et al.* compared over 360 CD patients with matched controls and suggested that CD seems to be associated with an increased risk of cardiovascular outcome. A few years later, Ludvigsson *et al.* published a large, nationwide cohort study and showed that increased risk of stroke in CD patients occurs in the first year after diagnosis, but it is no more relevant after 5 years of follow up [10]. These authors also showed no differences between children and adults with CD with respect to the risk of stroke.

Prompted by the ambiguity of the aforementioned studies, we decided to look into particular risk factors that might lead to increased prevalence of atherosclerosis (AS) in CD patients. In this review we present the current data and research on the risk factors of AS with respect to CD.

## 2. Inflammation in Celiac Disease and Atherosclerosis

Inflammation plays a key role in pathogenesis of AS, and it is considered that pro-inflammatory immune cells activated in CD could affect the development of atherosclerotic lesions. CD is a chronic immune-mediated inflammatory bowel disorder with an autoimmune component, in which gluten and related proteins induce specific CD4+ T lymphocytes [11]. Those cells are activated in lamina propria of small intestine by gluten derived peptides bound to DQ2 and/or DQ8 molecules located on the surface of antigen-presenting cells. Deamidation of glutamine to glutamic acid residues by tissue transglutaminase (tTG) causes negative polarity of gluten peptides, which significantly increases the gluten peptide binding to HLA class II molecules. Gluten-sensitive T lymphocytes produce a cascade of cytokines characterized by interferon (IFN)-gamma and to a lesser extend interleukin (IL)-6 and tumor necrosis factor (TNF)-alfa. Overexpression of pro-inflammatory cytokines stimulates other cells, including macrophages and fibroblasts, which potentiate the inflammatory response. Inflammation leads to destruction of the architecture of the small intestine finally resulting in crypt hyperplasia and villous atrophy. Inflammatory responses of the mucosal immune system in CD patients are not limited to the small intestine. T lymphocytes specific to tTG-deamidated gluten peptides were found in peripheral blood of untreated CD patients [12]. These patients presented a memory cell phenotype and expressed β7 integrin as a marker of gut homing. Analysis of cytokine profile showed that circulating T lymphocytes and macrophages secrete IFN-gamma and IL-6 [13]. Thus, pro-inflammatory cells found in blood of CD patients could activate processes inducing AS. Cytokine-stimulated endothelial cells start to express adhesion molecules and initiate the binding of inflammatory T lymphocytes that are found in the early AS plaques [14]. This hypothesis was confirmed by adoptive transfer experiments, which proved a pathogenic role for CD4+ T lymphocytes in mouse AS models [15]. In AS patients, the presence of increased percentages of CD4+ T lymphocytes producing IFN-gamma and TNF-alfa in complicated plaques in comparison with uncomplicated ones, supports the concept of the key role of activated T cells in the progression of atherosclerotic lesions [16].

Recently, IL-17 produced by T helper 17 cells (Th17) was proposed to be involved in the pathogenesis of both AS and CD. IL-17A and Th17 cells infiltrating atherosclerotic plaques were observed in AS patients [17]. Lim *et al.* [18] hypothesized that proatherogenic factors, especially oxidized low-density lipoproteins (ox-LDL), induce Th17 cell activity in AS. Upregulation of Th17 activity was also found in mucosa of active CD, but mucosal gliadin-specific Th17 cells presented a dual role as they produce pro-inflammatory cytokines (IL-17, IFN-gamma), mucosa-protective IL-22, and regulatory transforming growth factors (TGF)-beta, which is able to modulate IL-17 production by T cells [19].

Although gluten is the main external trigger of CD, its intake does not fully explain pathogenesis of the disease. Lahdenperä *et al.* [20] presented that GFD, which improves mucosal lesions, did not correct the increased activation of the IFN-gamma signaling pathway related markers. Thus, other environmental factors are thought to be involved in the initiation and progression of pathological processes in CD. Recent findings have implicated the gut microbiota as a contributor of autoimmune and inflammatory diseases [21]. Imbalance in the composition of gastrointestinal microbiota, characterized by increased number of *Bacteroides* species and the reduction of *Bifidobacterium* species, was associated with CD [22]. *In vitro* assays showed that these altered microbiota and some *Enterobacteria* isolated from CD patients, could activate pro-inflammatory pathways [23]. Alterations in the intestinal microbiota are also involved in the pathogenesis of several metabolic disorders, including obesity, diabetes and AS [24]. The gut metagenome of symptomatic AS patients was enriched in genes encoding peptidoglicans, *i.e.*, molecules which may contribute to AS by priming the pro-inflammatory innate immunity, whereas genes involved in synthesis of anti-inflammatory and anti-oxidants (β-carotene) were depleted [25]. Thus, the altered gut microbiota in AS may produce lower amounts of anti-oxidants.

Oxidative modification of LDL plays a key role in the pathogenesis of AS, and it was shown that ox-LDL initiate and accelerate the development of the early atherosclerotic lesion [26]. Oxidative stress, caused by an imbalance of antioxidants and oxidants, leads to an oxidation of the lipid membranes. Ox-LDL have a direct toxic impact on the endothelial and smooth muscle cells [20]. It has been demonstrated, that gliadin sequence contains regions, which not only induce the release of the pro-inflammatory cytokines, but also trigger oxidative stress [27]. One of the gliadin peptides, P31–43, was shown to accumulate in lysosomes and thus increase level of the free radicals [28,29]. Based on these findings the increased risk of AS in untreated CD patients could be explained by the gliadin peptides contribution in the oxidative modification of LDL, but still the impact of the gut microbiota in CD on the potential risk of cardiovascular disease is unclear.

## 3. Body Mass Index in Patients with CD

The clinical presentation of coeliac disease has changed in the last two decades and there is an increasing prevalence of overweight or even obese CD patients, both at the time of the diagnosis and/or after months or years the GFD was introduced [30,31]. Overweight and obesity are an important risk factors of metabolic syndrome and cardiovascular disease. There are several studies concerning the increased Body Mass Index (BMI) in adult cohorts of patients with CD. Dickey *et al.* [32] showed overweight (according to BMI ≥ 25) in 34% of 50 newly diagnosed adult patients. In this group, 3 out of 17 patients were obese and presented with BMI ≥ 30. Almost a decade later, Dickey presented results of another study, where a cohort of 371 patients observed for a period of 10 years was evaluated in terms of BMI [33]. Even though mean BMI in this group was in normal range (24.6 kg/m^2^), 39% of patients were overweight, including 13% in the obese range. Authors evaluated patients according to the coherence to the GFD and showed that 81% patients compliant to the GFD gained weight during the period of 2 years of observation, including 82% already overweight patients. A nationwide study of Ukkola and coworkers published in 2012 revealed that in the population of newly detected adults with CD (*n* = 698) over one third of patients were overweight or obese (28% and 11%, respectively) [34]. This trend was consistent with later investigations [35,36], however Kabbani *et al.* [36] showed coeliac cohort to be significantly less likely to be obese or overweight when compared to the regional (American) population data. Similar results were presented by Cheng *et al.* [37]; they showed that GFD has a beneficial impact on CD patients resulting in the weight increase in underweight patients, but they also showed that 54% of overweight and 47% of obese patients lost weight following the GFD. In the recent Dutch study on 80 untreated adult patients with CD, Wierdsma and colleagues showed overweight in 29% of patients, still a number of patients were malnourished or underweight (17% and 22%, respectively) [38].

Studies in young patients with CD show divergent results. Aurangzeb *et al.* [39] assessed a group of 25 children with CD and showed that over 20% of them were overweight. In contrast, less than 9% of these patients were malnourished. Another study of 150 children with CD showed less overweight and less obesity and more underweight in comparison to healthy controls (12% *vs.* 23.3% and 16% *vs.* 4.5%, respectively) [40]. A lot of data on the obesity of the pediatric CD patients come only from the case reports and therefore cannot be considered as a strong evidence [41].

Most of the presented studies showed an increase in BMI in patients with CD not only at the time of diagnosis, but even after GFD was introduced. This factor may play an important role in atherogenic effect and especially in younger patient may lead to rapid progress of the cardiovascular disease.

There are only a few studies concerning the prevalence of the metabolic syndrome in CD patients. In the study of Kabbani *et al.* [42], the authors aimed to determine the prevalence of the non-insulin dependent diabetes mellitus and metabolic syndrome (with hypertension as one of its criteria) in 840 CD patients, compared to 840 controls and 5514 individuals from the national population data (the NHANES population). They showed significantly lower prevalence of the metabolic syndrome in CD group. Moreover, West *et al.* [9], in a study of adult general population from Cambridge, showed that patients with undetected CD had a more favorable cardiovascular risk profile (in terms of lower cholesterol levels), significantly lower weight and diastolic blood pressure.

## 4. Gluten-Free Diet as a Potential CD Risk Factor

There are limited studies on the impact of gluten-free diet on cardiovascular disease’s risk factors. Moreover, the data on the lack of the adherence to the GFD can significantly carry weight to the results and lead to wrong conclusions. Few studies have shown that CD patients’ diet is overloaded with animal protein, energy and lipids, being at the same time insufficient in carbohydrates, fiber, iron and calcium [43,44]. Zanini *et al.* [45] showed significant increase in BMI and total cholesterol in a group of 715 CD patients adherent to GFD for 1 to 5 years. They also showed lowering of homocysteine and serum triglycerides comparing to baseline results, thus suggesting that the diet is unlikely to be atherogenic. In a recent study of German CD patients, no differences in the intake of energy and macronutrients in males were found. However, female patients showed a significantly higher fat intake, but lower carbohydrate consumption, as compared to the reference values. The average daily micronutrient intake of both, male and female CD patients, was significantly lower for vitamin B1, B2, B6, folic acid, magnesium and iron [46]. Some of the studies showed a nutritional safety of GFD in the first few years of treatment [47], but Bardella *et al.* [48] showed also the importance of the time of introducing GFD on the nutritional and bone status in CD patients.

With respect to the atherosclerosis risk factors, as mentioned above, GFD is insufficient in folate and some other micronutrients including vitamin B6. Hallert *et al.* [49] showed poor vitamin status in half of CD patients, particularly plasma folate, pyridoxal 5′-phosphate (active form of vitamin B6) and vitamin B12. The lowering of folate can result in elevated mean plasma homocysteine concentration and thus lead to atherosclerosis. Low level of folate in CD patients is however conflicting with the folate content in food products- cereal or whole-grain products contain less folates than fresh fruits, vegetables or soy, which are not restricted in GFD. Some countries fortify flour with folate therefore the sudden change in bread consumption should be followed with balanced folate ingestion or supplementation [50]. In some countries, gluten-free products are also fortified with folates [51].

In the majority of CD patients studied by De Marchi *et al.* [52] hyperhomocysteinemia was observed, which persisted after gluten withdrawal. De Marchi *et al.* [52] also studied carotid artery intima-media thickness (c-IMT), the marker of the non-invasive assessment of the early-stages of atherosclerotic vascular changes. Significant increase of c-IMT was found in the CD group compared to the controls; in that case, however, the introduction of GFD for 6–8 months resulted in a significant decrease of c-IMT in CD patients.

In untreated CD patients an impaired nutritional status is commonly seen [47]. It was shown that the level of vitamin D decreased at the time of the CD diagnosis, but it increased after one year of gluten withdrawal [47]. There is evidence of increased risk of cardiovascular diseases in adults with hypovitaminosis D, but for paediatric population it is scarce. Also, the evidence for a causal relation between vitamin D intake and development of disease was not found. In the latest consensus statement of ESPGHAN Committee on Nutrition on the vitamin D, it was concluded that more evidence is needed to make any recommendation on the effect of vitamin D supplementation to prevent cardiovascular diseases later in life [53].

Along with the recent trends of introducing GFD as a method of healthy life style or intentional weight loss, study of Soares *et al.* [54] appeared aimed to explain the mechanisms of gluten impact on obesity. In this mice study, authors showed the beneficial effects of GFD in reducing adiposity gain, inflammation and insulin resistance, without changes in food intake or lipid excretion [54]. Still, the impact of GFD on obesity requires further observations in human studies.

## 5. Cholesterol Profile in Patients CD

Serum level of total cholesterol is low in both diagnosed and undiagnosed CD patients [7,8,55,56]. This might be due to the malabsorpion, reduced cholesterogenesis, increased biliary secretion or high fecal elimination of cholesterol in not treated patients [57]. Lewis *et al.* [58] showed that total cholesterol is not only lower in CD patients comparing to healthy controls, but also it doesn’t increase after following a GFD for one year. Moreover, these authors observed an increase in HDL-cholesterol in CD patients following the treatment. Brar *et al.* [59] studied 132 CD patients strictly adherent to the GFD for a median of 20.5 months. They showed an increase in total cholesterol, as well as high-density lipoprotein (HDL), but not in low-density lipoprotein (LDL).

The aforementioned studies suggested that treatment with the GFD led to the improvement of the imbalanced lipid profile of the newly diagnosed or undiagnosed CD patients [58,59]. Thus, proper treatment with GFD seems to be an anti-atherosclerotic factor in CD patients.

## 6. CD and Type I Diabetes Mellitus

Coeliac disease is frequently associated with other autoimmune diseases such as type 1 diabetes mellitus (T1DM). Shared genetic background of these two and other autoimmune diseases has been demonstrated [60,61]. In T1DM, cardiovascular mortality is mostly pronounced in young people [62], but it is not known if it is increased with the coexistence with CD. Pitocco *et al.* [63] studied the mean carotid artery intima-media thickness (c-IMT) in patients with CD alone, both CD and T1DM and healthy controls. They showed increased c-IMT in CD patients in comparison to healthy controls, and the highest c-IMT in patients with both CD and T1DM. The data exist to support decreased endothelial damage and protective role of CD in diabetic patients due to lower status of some haemostatic factors against prothrombotic phase leading to vascular dysfunction [64]. In a study by Leeds *et al.* [65] the biochemical parameters and lipids’ profile, as well as the degree of neuropathy, retinopathy and nephropathy in adult T1DM patients with CD were analyzed. They showed worse glycemic control, as well as lower total cholesterol with lower HDL cholesterol and higher prevalence of microvascular complications in diabetic patients suffering additionally from CD. A recent study by Taler *et al.* [66] showed, however, that growth and measures of metabolic control did not differ among patients with T1DM and CD treated with GFD and those with T1DM alone. As the rate on non-adherence to the GFD seems to be much higher in diabetic patients than in CD-only patients (over 59% of CD-T1DM patients are not compliant to GFD [67]), the results may be biased so these data need further investigations.

## 7. Conclusions

In patients with chronic, lifelong diseases, a therapeutic approach should not only emphasize current treatment, but it should also predict and prevent later complications and concomitant diseases. There is a body of data published on cardiovascular complications in patients with CD, however conclusions of some studies are at odds with each other. For some studies, potential risk factors of atherosclerosis decline after gluten-free diet is introduced.

CD is unique among chronic diseases due the combination of three factors: it is associated with specific comorbidities, involves impaired nutrition, and specific diet is the only constant treatment. It can occur very early in life, therefore long-term health maintenance becomes an issue. Studies discussed in this article pinpoint characteristics of CD with respect to the potential risk factors that may be involved in the development of atherosclerosis and cardiovascular diseases. Thus far unsolved problems and areas to be addressed in the future include in particular:

1. long-term nutritional deficiencies on gluten-free diet, with hyperhomocysteinemia as a potential AS risk factor;
2. impact of the gut microbiota;
3. risk of cardiovascular complications in CD patients with co-occurrence of T1DM and other comorbidities;
4. long-term care and follow-up of CD patients with respect to the compliance to the GFD, preventing of nutrients deficiencies, early diagnosis of comorbidities.

The current practice guidelines regarding the follow-up of CD patients varies greatly among countries. We believe that a general long-term care program could reduce the risk of CD-associated complications.
